# An exploratory *in silico* analysis of bacteriocin gene clusters in the urobiome

**DOI:** 10.20517/mrr.2023.78

**Published:** 2024-03-27

**Authors:** Jennifer Jones, Craig P. Murphy, Roy D. Sleator, Eamonn P. Culligan

**Affiliations:** Department of Biological Sciences, Munster Technological University, Bishopstown, Cork T12 P928, Ireland.

**Keywords:** Urobiome, bacteriocins, antibiotic resistance, BAGEL4, bacteriocin gene cluster

## Abstract

**Background:** The role of the urobiome in health and disease remains an understudied area compared to the rest of the human microbiome. Enhanced culturing techniques and next-generation sequencing technologies have identified the urobiome as an untapped source of potentially novel antimicrobials. The aim of this study was to screen the urobiome for genes encoding bacteriocin production.

**Methods:** The genomes of 181 bacterial urobiome isolates were screened *in silico* for the presence of bacteriocin gene clusters using the bacteriocin mining tool BAGEL4 and secondary metabolite screening tool antiSMASH7*.*

**Results:** From these isolates, an initial 263 areas of interest were identified, manually annotated, and evaluated for potential bacteriocin gene clusters. This resulted in 32 isolates containing 80 potential bacteriocin gene clusters, of which 72% were identified as class II, 13.75% as class III, 8.75% as class I, and 5% as unclassified bacteriocins.

**Conclusion:** Overall, 53 novel variants were discovered, including nisin, gassericin, ubericin, and colicins.

## INTRODUCTION

The human microbiome and its role in health and disease have been at the forefront of scientific research in recent times^[1-4]^. To date, bacterial communities from numerous body sites have been screened, both *in silico* and *in vitro*, for antimicrobial compounds^[5-11]^. Recent advances in metaculturomics and metagenomic sequencing have led to the discovery and characterisation of the urobiome^[12,13]^, which represents a relatively understudied environment in terms of the diversity and novelty of bacteriocins encoded by this microbial community.

Bacteriocins are classified as ribosomally synthesised antimicrobial peptides, which are produced by bacteria as a defence mechanism against other bacteria present in the same environment^[5,14,15]^. Bacteriocins can display both narrow- and broad-spectrum bactericidal activity but are usually most effective against bacteria that are closely related to the producer strain^[6,8,16-18]^. While some bacteriocins are produced by Gram-negative bacteria^[19]^, the majority of bacteriocins characterised to date are produced by Gram-positive, lactic acid bacteria^[20-22]^. Bacteriocins have been divided into three classes: class I, also known as lantibiotics, are characterised based on the presence of the amino acid lanthionine or methyllanthionine as a result of post-translational modifications. The primary mode of action of class I bacteriocins is targeting the cell membrane^[14,20]^. Class II bacteriocins are smaller, thermostable peptides that can be further categorised into five subclasses^[23]^. They are classed as broad range antimicrobials and act by forming pores in the cell membrane. Class III bacteriocins are larger, heat-sensitive peptides that cause bacterial cell lysis^[5,14,20]^. In previous years, food preservation and other applications in the food industry were the primary focus of bacteriocin research^[15]^. More recently, this focus has shifted to antimicrobial resistance, and strategies to improve the treatment and control of antibiotic-resistant infections^[15]^, mainly centred on *in vivo* animal studies^[24]^. Bacteriocins have numerous desirable traits as antimicrobials, which make them particularly attractive alternatives to antibiotics, including low toxicity, high potency, and, most importantly, the ability to be effectively bioengineered^[18,23]^. Furthermore, a narrower activity spectrum than conventional antibiotics significantly reduces undesirable collateral damage to the commensal microbiota^[2,11,14]^.

*In silico* screening of bacterial genomes for bacteriocin production has significantly reduced both the time and cost of culture-based approaches for bacteriocin discovery^[25]^, with gene mining tools such as BAGEL4 and antiSMASH7^[26,27]^ enabling the rapid identification of bacteriocin gene clusters. BAGEL4 scans the bacterial genome for putative bacteriocin open reading frames (ORFs). It searches for the structural bacteriocin gene, but also takes advantage of the common structure of bacteriocin operons and scans the surrounding ORFs for possible accessory genes that encode immunity, transport, regulation, and modification proteins^[25,28,29]^. AntiSMASH7, on the other hand, uses set “rules” that identify core biosynthetic functions present within a genomic region to create a biosynthetic gene cluster (BGC). AntiSMASH7 combines different profile hidden Markov model “rules” to identify 81 different BGC types^[27]^.

**Figure 1:**
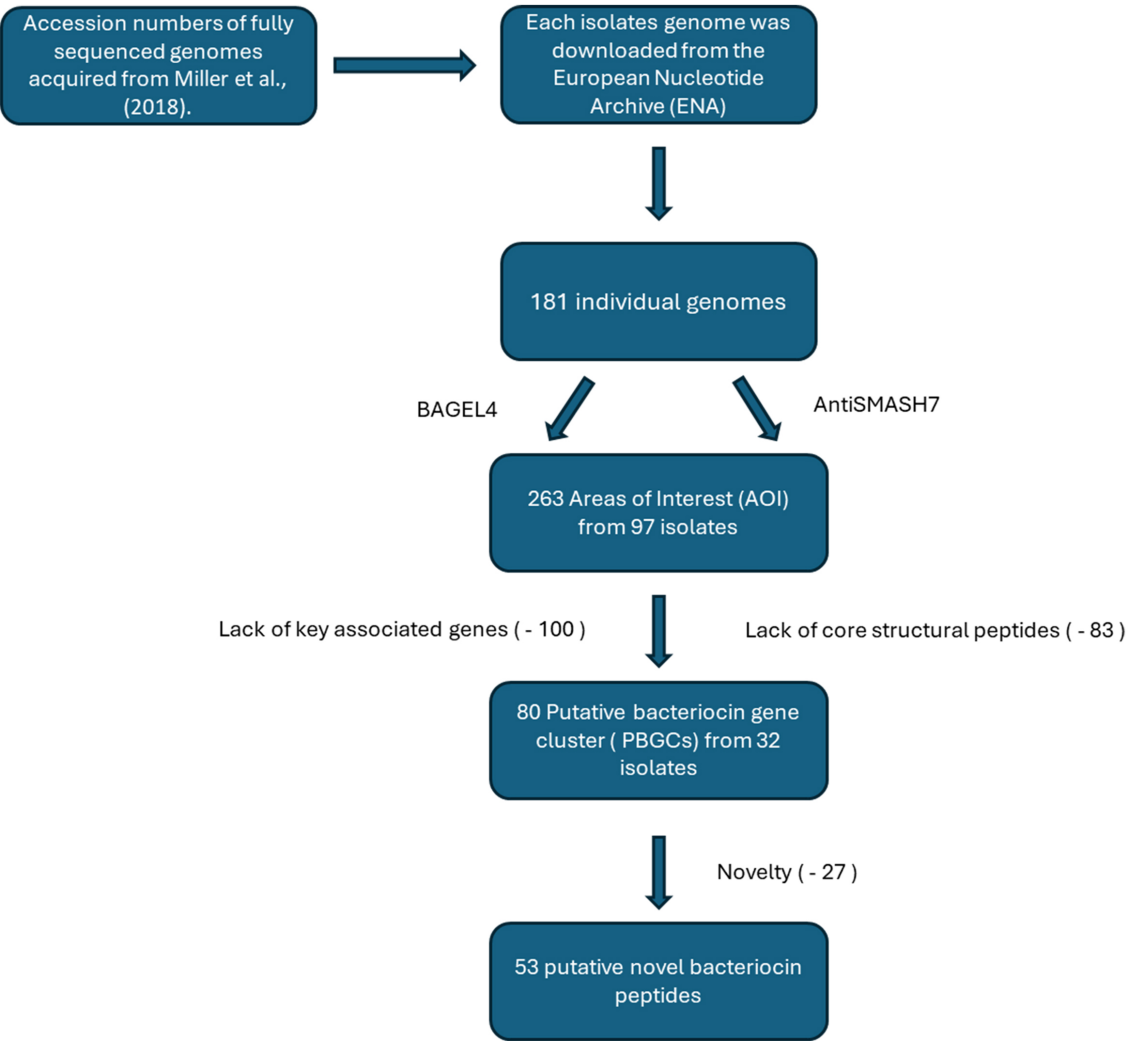
Flow chart of bacteriocin screening performed in this study.

The current study is, to the best of our knowledge, the first to screen the urobiome for genes encoding bacteriocin production. Herein, we screened 181 bacterial isolates previously isolated from the bladder^[30]^, with the primary objective to identify novel bacteriocin clusters in the urobiome [Figure 1]. Initially, 263 putative bacteriocin gene clusters were identified, highlighting the potential of the urobiome to host a diversity of bacteriocin producers.

## METHODS

### Data collection

The 181 fully sequenced genomes examined in this study were from urinary bacterial isolates, collected via catheter, previously isolated, sequenced, and assembled by Miller-Ensminger *et al.* (2018)^[30]^. Accession numbers were obtained [Supplementary Data], and each individual genome was downloaded from the European Nucleotide Archive (ENA) (https://www.ebi.ac.uk/ena/browser/home) in FASTA format.

### Initial screening

The bacteriocin mining tool BAGEL4 (http://bagel4.molgenrug.nl/index.php)^[26]^ was used in combination with antiSMASH7 (https://antismash.secondarymetabolites.org/#!/start)^[27]^ to identify and analyse putative bacteriocin gene clusters (PBGCs), using default parameters.

### Further analysis of individual gene clusters

Areas of interests (AOIs) identified by BAGEL4, predicted to be bacteriocins or associated with bacteriocin production, were investigated using BLASTP (https://blast.ncbi.nlm.nih.gov/Blast.cgi?PROGRAM=blastp&PAGE_TYPE=BlastSearch&LINK_LOC=blasthome)^[31]^. An AOI was considered to be a PBGC if it contained a structural core peptide and if it was surrounded by the key associated genes previously described in the literature, such as immunity, transport, leader cleavage, and a modification gene for post-translationally modified peptides^[9,29]^. To determine the degree of novelty of the PBGCs, the amino acid sequences for bacteriocin production were aligned against their closest characterised homologues, as indicated by BLASTP using the sequence alignment tool EMBL-EBI EMBOSS Needle (https://www.ebi.ac.uk/Tools/psa/) using the Needleman-Wunsch algorithm. Novelty was described when a difference of two or more amino acids was identified in the predicted bacteriocin sequence compared to previously characterised bacteriocins^[10]^. All amino acid sequences of the surrounding accessory proteins displayed > 95% identity to their predicted proteins, unless otherwise stated.

## RESULTS

### *In silico* screening for putative bacteriocin gene clusters

This study screened 181 bacterial urobiome isolates^[30]^ cultured from catheterised urine samples. Catheterised urine samples reduced the risk of cross-contamination from surrounding microbiomes (urethra, skin, vagina, *etc.*), and, as such, represent true urobiome/bladder isolates^[32]^. The initial screening using BAGEL4 and antiSMASH7 resulted in the identification of 263 AOIs [Supplementary Table 1]. BAGEL4 identifies the presence of AOIs within the genomes; however, this does not necessarily translate into functional peptide production for reasons including mutations, regulation, or target specificity.

In total, 263 AOIs were identified from 97 isolates across 35 genera, with 54 of the isolates predicted to produce more than one putative bacteriocin [Supplementary Table 1]. Further analysis revealed that 83 of the AOIs lacked a core structural peptide sequence. Of these, 72 of the identified bacteriocin operons contained the full complement of accessory genes necessary for bacteriocin production but appeared to lack the required core structural peptides. Thirty-six strains encoded sactipeptide gene clusters with no core peptide, and 16 strains encoded bottromycin but again lacked the structural core peptide from 25 different genera including *Actinomyces*, *Aerococcus*, *Bacillus*, *Fingoldia*, *Gordonia*, *Staphylococcus*, *Klebsiella*, *Leclercia*, *Morganella* and *Pseudomonas*. A possible explanation for this is that the BAGEL4 database may simply not contain the sequence homologues of the core peptides^[33]^. It has also been hypothesised that bacteriocin production can be spontaneously acquired in the microbiome by horizontal gene transfer and can be lost by deletion of biosynthetic genes as bacteriocin production is metabolically costly^[18,34]^. Similarly, 20 putative helveticin J peptides predicted by BAGEL4 were excluded from further analysis due to the lack of a core peptide. It is also noteworthy that, in some studies, helveticin J peptides are no longer classed as bacteriocins and are considered a distinct group of antimicrobials (called bacteriolysins)^[28]^.

Of the remaining 180 putative bacteriocin AOIs, 100 were determined to be lacking the key associated genes for bacteriocin production and, as such, were eliminated [Figure 1]. This resulted in 32 remaining isolates with 80 PBGCs that contained a structural core peptide and the associated accessory genes [Supplementary Table 2]. While they were removed based on the parameters set for this study, we do accept the possibility that these gene products may work in conjunction with other novel bacteriocins/bacteriocin-related genes encoded elsewhere on the genome^[33]^. Of the 80 remaining bacteriocins, 72% were identified as class II, 13.75% as class III, 8.75% as class I, and 5% as unclassified bacteriocins.

### Further analysis of PBGCs of particular interest

**Figure 2:**
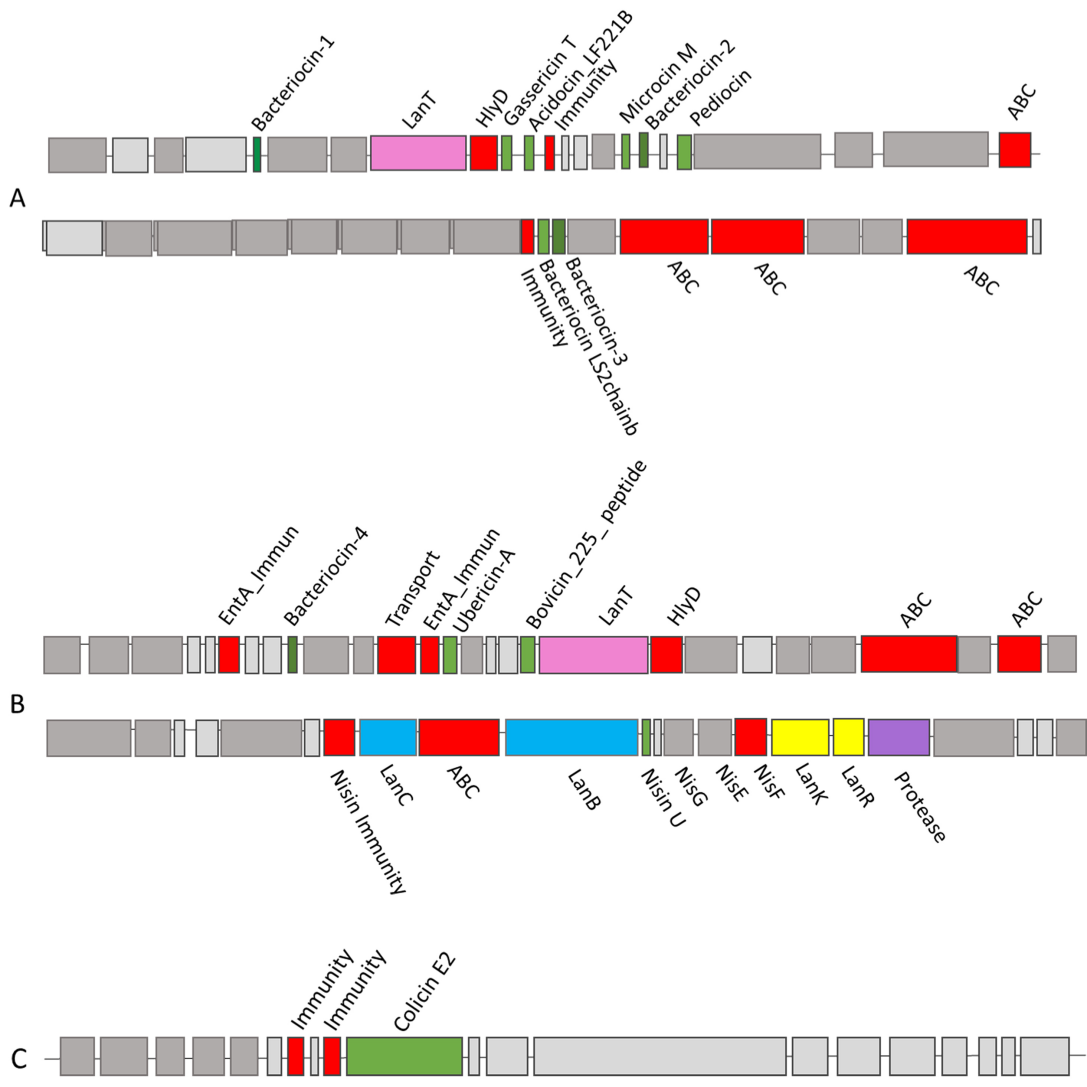
BAGEL4 outputs for urobiome isolates from (A) Lactobacillus gasseri UMB0099 depicting a putative microcin gene cluster and a class IIb gene cluster; (B) from Streptococcus macedonicus UMB0733 depicting a putative ubericin- A gene cluster and a putative novel nisin U gene cluster; and (C) from Proteus mirabilis UMB0315 depicting a putative active colicin bacteriocin gene cluster.

**Figure 3:**
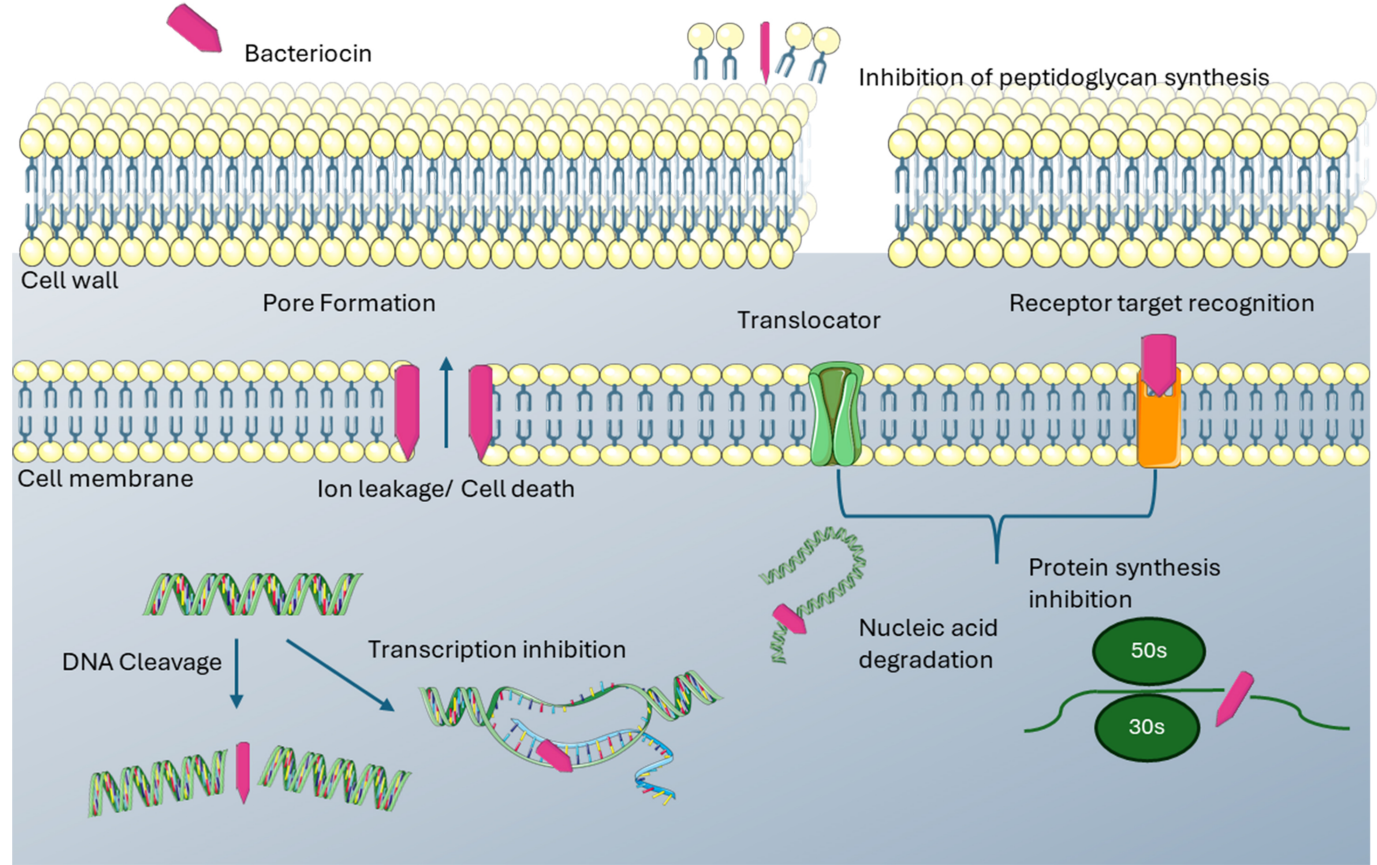
Mechanism of action of the putative bacteriocins identified in Table 1.

Based on BAGEL4, BLASTP, and EBI EMBOSS Needle analysis of the urobiome isolates, 53 putative bacteriocin hits were determined to be potentially novel [Supplementary Table 2]. Novelty in this case is taken as a core peptide with two or more amino acid differences compared to its closest characterised homologue^[10]^, or a 100% identity to previously uncharacterised bacteriocin with no other closely related characterised homologues. Three bacterial strains, *Lactobacillus gasseri* UMB0099, *Streptococcus macedonicus* UMB0733, and *Proteus mirabilis* UMB0315, were chosen for further analysis [Table 1, Figures 2 and 3].

**Table 1:** Abridged version of urobiome strains of interest which encode putative bacteriocin gene cluster(s) with surrounding accessory genes present

**BAGEL4 AOIs**	**Genome name**	**BLASTP result**	**Query cover**	**% identity**	**Alignment EMBOSS**	**Accession Number**
ACIDOCIN_LF22IB (GASSERICINK7B)	*Lactobacillus gasseri* str. UMB0099	Blp family class II bacteriocin [*Lactobacillus*]	100%	100%	100%	WP_003649213.1
BACTERIOCIN_LS2CHAINB*	*Lactobacillus gasseri* str. UMB0099	Blp family class II bacteriocin [*Ligilactobacillus salivarius*]	97%	47.76%	43.8%	WP_032495430.1
BOVICIN_225_PEPTIDE*	*Streptococcus macedonicus* str. UMB0733	Bacteriocin class II with double glycine leader peptide [*Streptococcus infantarius subsp. Infantarius*]	100%	93.5%	95.9%	MCO4620482.1
COLICIN*	*Proteus mirabilis* str. UMB0315	Colicin-E2 [*Proteus mirabilis*]	99%	99%	99.1%	AWF41001.1
COMC; L_BIOTIC_TYPEA; BACTERIOCIN_IIC*	*Streptococcus macedonicus* str. UMB0733	Bacteriocin [*Streptococcus gallolyticus*]	100%	78%	78%	WP_114317773.1
GASSERICIN_T	*Lactobacillus gasseri* str. UMB0099	Blp family class II bacteriocin [*Lactobacillus paragasseri*]	100%	100%	100%	WP_049159833.1
GGMOTIF; BACTERIOCIN_IIC;	*Lactobacillus gasseri* str. UMB0099	Blp family class II bacteriocin [*Lactobacillus*]	100%	100%	100%	WP_101890487.1
L_BIOTIC_TYPEA; BACTERIOCIN_IIC*	*Lactobacillus gasseri* str. UMB0099	Lactacin F inducer peptide precursor [*Lactobacillus johnsonii*]	100%	84%	92%	WP_260307981.1
MICROCIN_M*	*Lactobacillus gasseri* str. UMB0099	Bacteriocin [*Lactobacillus*]	100%	100%	100%	WP_101890486.1
NISIN_U*	*Streptococcus macedonicus* str. UMB0733	Gallidermin/nisin family lantibiotic [*Streptococcus suis*]	100%	100%	75%	WP_228478826.1
PEDIOCIN*	*Lactobacillus gasseri* str. UMB0099	Bacteriocin immunity protein [*Lactobacillus*]	100%	100%	100%	WP_101890489.1
UBERICIN_A*	*Streptococcus macedonicus* str. UMB0733	Blp family class II bacteriocin [*Streptococcus*]	93%	100%	93.5%	WP_003066580.1
UNIDENTIFIED BACTERIOCIN*	*Lactobacillus gasseri* str. UMB0099	Bacteriocin [*Lactobacillus*]	100%	100%	100%	WP_049160225.1

A full summary of all 53 novel urobiome strains which encode putative bacteriocin gene cluster(s) is available in Supplementary Table 2. Putative bacteriocin hits are presented with their closest homologues as identified through BLASTP analysis and alignment using EBI EMBOSS Needle, with an asterisk (*) representing the bacteriocins that are potentially novel by either differing by two or more amino acids or matching to a reported but previously uncharacterised bacteriocin. AOIs: Areas of interests.

#### Analysis of selected novel PBGCs identified among Lactobacillus species

*Lactobacillus* strains were identified most often (54%) when analysing urobiome isolates for novel bacteriocin production. *Lactobacillus* is one of the most common bacterial species isolated from the urobiome, particularly in women^[13,28,35]^, and is thought to play a protective role within the urobiome^[36]^. *Lactobacillus* species are well-characterised bacteriocin producers^[22]^ and have been highlighted for their potential applications in medicine, veterinary medicine, and the food industry as effective alternatives to antibiotics and food preservatives^[37]^. Additionally, many lactic acid bacteria (LAB) and their products are generally recognised as safe (GRAS status) by the FDA^[37,38]^.

*Lactobacillus gasseri* UMB0099 was isolated by Miller-Ensminger *et al.* (2018)^[30]^ from a urine sample collected by catheter from a patient suffering from an overactive bladder (OAB). BAGEL4 analysis revealed that this strain encoded nine putative bacteriocin peptides in total. One was removed as it lacked the associated production proteins. The remaining eight included three “generic” bacteriocins (dark green peptides; bacteriocin 1-3), gassericin T, acidocin_LF221B, microcin M, pediocin, and a bacteriocin LS2 chain b peptide [Figure 2A]. The gassericin T, acidocin_ LF221B (gassericin K7B), and bacteriocin-2 putative core peptides [Figure 2A] all represent 100% identity to previously characterised Blp class II bacteriocins^[39,40]^. Blp bacteriocin gene clusters are known to encode multiple bacteriocin-like peptides^[39]^ which are secreted by ATP binding cassette (ABC) transporter proteins, similar to the PBGC identified in this study. Both gassericin and acidocin have been previously studied for their ability to prevent the growth of *Staphylococcus aureus* from mastitic milk and used as food preservatives and therapeutic agents for mastitis^[41]^. While the putative pediocin gene was predicted by BAGEL4 to encode a core bacteriocin peptide, once blasted, this was determined to exhibit 100% identity to an immunity protein and, thus, is likely mis-annotated and is not a core bacteriocin peptide. The putative cluster also contained a *LanT* gene, cleavage/export ABC transporter, HylD protein, and an immunity protein. The presence of all the accessory genes suggests functionality of the PBGC, although experimental validation is required for confirmation^[41]^.

Of interest in this PBGC are the microcin M and bacteriocin-1. The microcin M on this cluster exhibits 100% identity to an uncharacterised bacteriocin, not a microcin. While BLASTP analysis did identify one uncharacterised bacteriocin, unlike other putative hits, it appears to have no other homologues. Microcins are typically produced by Gram-negative *Enterobacteriaceae* family^[42]^ and have not been found before in Gram-positive bacteria. Therefore, with no other closely related hits, it can be inferred that it is either misannotated or is an uncharacterised novel bacteriocin. Bacteriocin-3 revealed a novel lactacin F precursor protein, with 84% identity to an uncharacterised lactacin F precursor isolated from *Lactobacillus johnsonii*. This is similar to other studies that have found multi-bacteriocin producing LAB strains that also encoded gassericin and acidocin on the same operon. These previously characterised bacteriocins have been shown to inhibit enteric bacteria and retain activity in colon conditions *in vitro*^[43]^. Production of multiple bacteriocins by a single strain is a desirable trait and can expand their spectrum of inhibition against different pathogenic strains. For example, Jiang *et al.* found synergistic activity, *in vitro*, of a two-peptide bacteriocin against a pathogenic *Salmonella* strain^[44]^.

The putative class IIb PBGC shown in Figure 2A contained two core bacteriocin peptides: bacteriocin LS2 chain b and an unidentified bacteriocin. After analysis using BLASTP and EMBI-EBI EMBOSS Needle, the bacteriocin LS2 chain b showed a 43.8% identity to BIp family class II bacteriocin isolated from *Ligilactobacillus salivarius*^[40]^, demonstrating a potentially novel variant of a class II bacteriocin. Similarly, the unidentified bacteriocin exhibited 100% identity to an uncharacterised bacteriocin; however, further investigation using BLASTP and EBI EMBOSS Needle alignment showed 91.1% identity to a previously characterised BIp family class II bacteriocin. These two putative BIp peptides were also found in *Lactobacillus gasseri* strain UMB0056. Among the *Lactobacillus* urobiome isolates, numerous strains encoded putative novel BIp class II bacteriocins with all the necessary accessory genes required for production present. Strains encoding novel class II two peptide bacteriocins include *Lactobacillus crispatus* strains UMB0040, UMB0803, UMB0805, UMB0044, UMB1398 and *Lactobacillus rhamnosus* UMB0004. Bacteriocins from LAB are of particular interest, with some already approved by the FDA for their use in food preservation, including nisin and pediocin^[38]^. Importantly, LAB bacteriocins have shown promise in inhibiting human pathogens in both *in vitro* studies against *Pseudomonas aeruginosa*^[45]^, uropathogenic *E.coli*^[46]^, and *Candida spp.*^[47]^ and in *in vivo* studies against *Gardnerella vaginalis*^[48]^, and in trials using bacteriocin-producing probiotics against bacterial vaginosis and UTIs^[49]^. Novel variants of these bacteriocins can aid in the treatment/prevention of infections, further highlighting the importance of *in silico* screening studies.

#### Analysis of various novel PBGCs identified from Streptococcus macedonicus

A study by Hilt *et al.* found *Streptococcus* to be a prevalent genus within the healthy female urobiome^[50]^. *Streptococcus* species produce many well-characterised bacteriocins^[51]^ such as salivaricin, streptolysin, and mutacin^[52]^, but none to date have been associated with the urobiome. *Streptococcus macedonicus* has previously been linked to bacteriocin production (macedocin) in dairy fermentations^[53]^. *S. macedonicus* UMB0733 was collected by catheter from a participant with no urinary symptoms or diseases^[30]^. BAGEL4 analysis on this strain identified multiple PBGCs.

The PBGC shown in Figure 2B was initially identified by BAGEL4 as an ubericin A bacteriocin gene cluster. Numerous *Streptococcus uberis* strains, producing multiple bacteriocins such as uberolysin A, ubericin A, and the lantibiotic, nisin U, have shown *in vitro* activity against different mastitis-inducing pathogens^[54]^. Further analysis of all three putative bacteriocin hits using BLASTP and EMBI-EBI EMBOSS Needle identified novel variants of class II bacteriocin peptides. The first bacteriocin hit shared 78% identity with an uncharacterised bacteriocin isolated from *Streptococcus gallolyticus*. The ubericin A hit shared 93.5% identity with a BIp family class II bacteriocin previously characterised by Dawid *et al.*^[39]^.

Finally, the bovicin 225 peptide shared 93.4% identity with a previously characterised class II bacteriocin with a double-glycine leader peptide isolated from *Streptococcus infantarius*. Alongside the three core peptides, two EntA immunity proteins were present on the genome with LanT, HlyD, and two ABC transporters. With three putative novel bacteriocins and all the accessory genes necessary for production, it can be inferred that this is a novel variant of class II bacteriocin gene cluster. Figure 2B shows a novel variant of the bacteriocin nisin U which displayed 75% identity to a gallidermin/nisin family lantibiotic previously characterised by Christ *et al.*^[55]^. With all eleven accessory genes present on the gene cluster [Figure 2B], it can be assumed to be an active variant of nisin U^[56]^.

#### Analysis of novel Colicin PBGCs identified from Proteus mirabilis

*Proteus mirabilis* UMB0315 [Figure 2C] was collected by catheter from a participant with symptoms of an overactive bladder^[30]^. BAGEL4 analysis identified a novel colicin bacteriocin gene cluster. Colicins, which are usually produced by *E. coli*, are among the most well-studied bacteriocins and are effective antimicrobials against other *E. coli* and *Enterobacteriaceae* strains^[57,58]^. The colicin of interest in this study was a novel variant produced by *Proteus mirabilis*. To date, it appears that colicins produced by *Proteus* have not been extensively characterised, but a crude bacteriocin extract from *Proteus mirabilis* has been described for its colicin-like antibiofilm properties^[59]^.

The PBGC, shown in Figure 2C, was identified by BAGEL4 as a colicin E2 bacteriocin gene cluster. Further analysis of the bacteriocin core peptide using BLASTP and EMBI-EBI EMBOSS demonstrated a novel variant of a colicin E2 peptide sharing 99.5% identity with six amino acid differences at positions 1, 57, 59, 86, 128, and 188. With both immunity proteins also present [Figure 2C], this suggests that this novel colicin variant is one of the first predicted to be produced by a *Proteus* species. Colicins have been previously highlighted for their antimicrobial potential by coating catheters to inhibit colonisation by UTI-causing pathogenic bacteria^[60]^. Colicins have numerous favourable properties such as the low concentrations needed for antimicrobial activity and also their specificity in killing, making them desirable antimicrobials that inflict limited collateral damage on the commensal microbiota^[61]^. Other putative colicin clusters identified in this study were found among the following urobiome strains: *Citrobacter murliniae* (UMB1094), *Escherichia fergusonii* (UMB0727, UMB0901, UMB0900, UMB0789), *Morganella morganii* (UMB1297) and *Pseudomonas aeruginosa* (UMB0710). New bacteriocins that have recently obtained GRAS status from the FDA for their use in food include colicins and colicin-like peptides (salmocins from *Salmonella*)^[58]^, further highlighting the utility of *in silico* screening studies to aid in the discovery of novel bacteriocins.

## DISCUSSION

In conclusion, previous *in silico* screening techniques have successfully identified bacteriocin gene clusters in the human microbiome^[5,6,9-11,33]^. However, given that urobiome research is in its nascent stages, investigation of its bioactive products has remained relatively understudied to date. This *in silico* analysis highlights the overall bacteriocin production ability of the urobiome. Bacteriocin production is a highly regulated process and requires specific environmental conditions, which complicates *in vitro* screening for bacteriocins. *In silico* screening, on the other hand, has allowed the rapid identification of bacteriocins without the restrictions of *in vitro* screening^[10,28]^. However, it is important to note that *in silico* screening is limited by the need for comparison to previously characterised bacteriocins, which can lead to completely novel bacteriocin gene clusters being missed^[28,33]^. Furthermore, such *in silico* screens are based solely on inference and can only be definitively verified by *in vitro* and or *in vivo* follow-up analysis. Notwithstanding, the current study determined that 19.33% (35/181) of strains isolated from the urobiome encoded one or more potentially active bacteriocin peptides*.* Despite these limitations, such studies remain an important first step in identifying novel bacteriocins. Bacteriocins have been isolated from a variety of microbiomes, demonstrating antimicrobial activity against clinically relevant pathogens. Bacteriocins have been used to target pathogens both *in vitro*^[45-47,54,62]^ and *in vivo*^[48,63]^, exerting probiotic effects^[49,64]^, inhibiting biofilm formation^[60]^, and resensitising resistant bacterial strains to antibiotics^[65]^, while demonstrating limited cytotoxic effects on the commensal healthy microbiomes. It is hoped that the identification of bacteriocins from untapped niches such as the urobiome can aid in the transition into use in clinical settings to control infections.

Overall, this study identified 53 putative novel bacteriocin peptides that have not been previously characterised, suggesting a high degree of novelty and diversity of bacteriocin production within the urobiome. These results indicate that the urobiome represents a comprehensive, and relatively untapped, source of novel antimicrobials, some of which might well find application in the control of antibiotic resistant infections*.*

## DECLARATIONS

### Availability of data and materials

The accession numbers for all genomes used for analysis in this manuscript can be found in Supplementary Data. All BAGLE4 and antiSMASH data can be found in Supplementary Tables 1 and 2.

### Copyright

© The Author(s) 2024.
