# Supplementary material for: An exploratory in silico analysis of bacteriocin gene clusters in the urobiome
Source: Microbiome Res Rep. 2024 Mar 27;3(2):24. doi: 10.20517/mrr.2023.78 (PMC11153084; doi:10.20517/mrr.2023.78)
Supplement: Supplementary file 2 [file mrr-3-2-24-SupplementaryMaterials.pdf]

Jones *et al. Microbiome Res Rep* 2024;3:24
DOI: 10.20517/mrr.2023.78

Microbiome Research Reports

Supplementary Materials

An exploratory in silico analysis of bacteriocin gene clusters in the urobiome

Jennifer Jones, Craig P. Murphy, Roy D. Sleator, Eamonn P. Culligan

Department of Biological Sciences, Munster Technological University, Bishopstown, Cork T12 P928, Ireland.

Correspondence to: Dr. Eamonn P. Culligan, Department of Biological Sciences, Munster Technological University, Bishopstown, Cork T12 P928, Ireland. E-mail: Eamonn.culligan@mtu.ie

Supplementary Table 1. Preliminary antimicrobial prediction by BAGEL4 and antiSMASH7 for the bacterial isolates from the urobiome

ISOLATE	BAGEL4					ANTISMASH7		
						Antibiotic	Antimicrobial peptide	Other
ACINETOBACTER SEPTICUS STR. UMB1319	-	-	-	-	-	-	-	NI- siderophore, Arylpolyene
ACTINOBACULUM SCHAALII STR. UMB0086	-	-	-	-	-	-	RiPP-like	Type III polyketide synthase (T3PKS)
ACTINOBACULUM SCHAALII STR. UMB0063	-	-	-	-	-	-	-	T3PKS
ACTINOBACULUM URINALE STR. UMB0759	-	-	-	-	-	-	-	T3PKS
ACTINOMYCES EUROPAEUS STR. UMB0652	-	-	-	-	-	-	-	-
ACTINOMYCES GRAEVENITZII STR. UMB0286	Sactipeptide	-	-	-	-	-	-	RRE-element containing cluster
ACTINOMYCES HOMINIS STR. UMB0859	LAPs	-	-	-	-	-	LAP, RiPP-like	-
ACTINOMYCES NAESLUNDII STR. UMB0181	Lasso peptide	Linocin M18	Sactipeptide	-	-	Thiopeptide	RiPP-like	RRE-element containing cluster

ACTINOMYCES NEUII STR. UMB0402	-	-	-	-	-	-	-	-
ACTINOMYCES NEUII STR. UMB0125	-	-	-	-	-	-	-	-
ACTINOMYCES NEUII STR. UMB0138	Sactipeptide	Microbisporicin	-	-	-	-	Lanthipeptide-class-I	-
ACTINOMYCES NEUII STR. UMB0918	Sactipeptide	-	-	-	-	-	-	-
ACTINOMYCES ODONTOLYTICUS STR. UMB0018	-	-	-	-	-	-	-	-
ACTINOMYCES SP. STR. UMB0183	Lactococcin	LAPs	Linocin	Sactipeptide	-	Thiopeptide	RiPP-like (3), LAP	-
ACTINOMYCES SP. STR. UMB0731	Linocin	Sactipeptide	-	-	-	-	RiPP-like, Lanthipeptide-class-III, LAP	-
ACTINOMYCES TURICENSIS STR. UMB0250	Thiopeptide	-	-	-	-	Thiopeptide	-	-
ACTINOMYCES UROGENITALIS STR. UMB0319	Sactipeptide	-	-	-	-	-	-	-
AEROCOCCUS CHRISTENSENII STR. UMB0844	Sactipeptide	-	-	-	-	-	-	-
AEROCOCCUS SANGUINICOLA STR.	Sactipeptide	Lichenicidin VK21A1	-	-	-	-	Lanthipeptide-class-II	-

UMB0139								
AEROCOCCUS URINAE STR. UMB0088	-	-	-	-	-	-	-	T3PKS
AEROCOCCUS URINAE STR. UMB0080	-	-	-	-	-	-	-	T3PKS
AEROCOCCUS URINAE STR. UMB0126	-	-	-	-	-	-	-	T3PKS
AEROCOCCUS URINAE STR. UMB0232	-	-	-	-	-	-	-	T3PKS
AEROCOCCUS URINAE STR. UMB0072	-	-	-	-	-	-	-	T3PKS
AEROCOCCUS URINAE STR. UMB0072	-	-	-	-	-	-	-	T3PKS
AEROCOCCUS VIRIDANS STR. UMB0240	-	-	-	-	-	-	-	-
ALLOSCARDOVIA OMNICOLENS STR. UMB0064	-	-	-	-	-	-	RiPP-like	T3PKS
ALLOSCARDOVIA OMNICOLENS STR. UMB0006	-	-	-	-	-	-	RiPP-like	T3PKS
ANAEROCOCCUS HYDROGENALIS STR. UMB0204	Sactipeptide	-	-	-	-	-	Ranthipeptide	-
ANAEROCOCCUS OCTAVIUS STR. UMB0119	Sactipeptide (2)	Propioncin_SM1	-	-	-	Equibacti n	-	RRE-element containing cluster

PSEUDOGLUTAMICIBACTER ALBUS STR. UMB0722	-	-	-	-	-	-	-	Beta lactone
ARTHROBACTER CUMMINSII STR. UMB0715	-	-	-	-	-	-	-	Beta lactone
BACILLUS ARYABHATTAI STR. UMB0500	Sactipeptide	UViB	-	-	-	-	-	T3PKS, phoshonate, NI-siderophore, CDPS, Terpene (3)
BACILLUS CIBI STR. UMB0893	Sactipeptide (2)	COMX3	Sonorensin	Lasso - peptide	-	-	Lasso - peptide, LAP	Terpene, NI - siderophore, T3PKS
BACILLUS DRENTENSIS STR. UMB0728	-	-	-	-	-	-	-	Terpene (4), T3PKS
BACILLUS SP. STR. UMB0899	LAPs	COMX3	Sonorensin	Sactipeptide	-	-	LAP (2)	Terpene (2), T3PKS, NI-siderophore
BIFIDOBACTERIUM BREVE STR. UMB0915	-	-	-	-	-	-	-	-
BIFIDOBACTERIUM BREVE STR. UMB0089	-	-	-	-	-	-	-	-
BIFIDOBACTERIUM LONGUM STR. UMB0788	-	-	-	-	-	-	-	-
BRACHYBACTERIUM FAECIUM STR.	Microbispori cin	Sactipeptide	Lanthipeptid e- Class-I	-	-	-	Lanthipeptide-Class-I, RiPP-	-

UMB0905							like	
BREVIBACTERIUM LUTEOLUM STR. UMB0680	Sactipeptide	-	-	-	-	-	-	NAPAA, Terpene, NAGGN, Tropodithietic-acid
BREVIBACTERIUM SP. STR. UMB0680	Sactipeptide	-	-	-	-	-	-	NAPAA, Terpene, NAGGN, Tropodithietic-acid
BREVIBACTERIUM RAVENSPURGENSE STR. UMB0426	-	-	-	-	-	-	-	Terpene, Ectoine
BREVIBACTERIUM PAUCIVORANS STR. UMB1301	-	-	-	-	-	-	NRPS	Terpene, NAPPA
CAMPYLOBACTER UREOLYTICUS STR. UMB0112	Sactipeptide	-	-	-	-	-	-	Arylpolyene
CITROBACTER MURLINIAE STR. UMB1094	Colicin	Colicin-10	Bottromycin	-	-	Thiopeptide	NRP-metallophore	-
CORYNEBACTERIUM SP. STR. UMB0338	-	-	-	-	-	-	NRP-metallophore	Terpene, T3PKS, NAPAA
CORYNEBACTERIUM SP. STR. UMB0042	-	-	-	-	-	-	NRP-metallophore	Terpene, T3PKS, NAPAA,

								aminopolycarboxylic-acid
CORYNEBACTERIUM AURIMUCOSUM STR. UMB1300	-	-	-	-	-	-	RiPP-like, NRP, NRP-metallophore	Terpene, T1PKS, NAPAA (2), aminopolycarboxylic-acid
CORYNEBACTERIUM SP. STR. UMB0043	-	-	-	-	-	-	-	Terpene, NAPAA, T1PKS
CORYNEBACTERIUM COYLEAE STR. UMB0147	-	-	-	-	-	-	-	Terpene, NAPAA, T1PKS
CORYNEBACTERIUM COYLEAE STR. UMB0989	-	-	-	-	-	-	-	NI- siderophore, Terpene, NAPAA, T1PKS
CORYNEBACTERIUM KROPPENSTEDTII STR. UMB0869	-	-	-	-	-	-	NRP-metallophore, NRPS (2)	Terpene, T3PKS, aminopolycarboxylic-acid
CORYNEBACTERIUM SP. STR. UMB0763	-	-	-	-	-	-	-	T1PKS, Terpene
CORYNEBACTERIUM SP. STR. UMB0243	-	-	-	-	-	-	-	T1PKS, Terpene, NAPAA
CORYNEBACTERIUM TUSCANIENSE STR. UMB0792	-	-	-	-	-	-	-	T1PKS, Terpene, Ectoine
CORYNEBACTERIUM XEROSIS STR. UMB0908	Sactipeptide	-	-	-	-	-	-	Terpene (2), T1KPS, NAPAA
DERMABACTER HOMINIS STR. UMB0490	-	-	-	-	-	-	-	T3PKS

DOLOSICOCCUS PAUCIVORANS STR. UMB0852	-	-	-	-	-	-	-	T3PKS
DOLOSICOCCUS PAUCIVORANS STR. UMB0860	-	-	-	-	-	-	-	T3PKS
DOLOSICOCCUS PAUCIVORANS STR. UMB0860	-	-	-	-	-	-	-	T3PKS
ENTEROBACTER AEROGENES STR. UMB0295	-	-	-	-	-	Lankacidin C	RiPP-like, NRPS	Arylpolyene
KLEBSIELLA AEROGENES STR. UMB0295	-	-	-	-	-	Lankacidin C	RiPP-like, NRPS	Arylpolyene
ENTEROCOCCUS FAECALIS STR. UMB0891	Cytolysin_Clyls	Enterocin_96	-	-	-	-	Cyclic lactone autoinducer, lanthipeptide-Class-II	RRE-element containing cluster
ENTEROCOCCUS FAECALIS STR. UMB0048	Cytolysin_ClyLs, Cytolysin_ClyL1	-	-	-	-	-	Lanthipeptide-Class-II	-
ESCHERICHIA COLI STR. UMB0789	Colicin	Microcin	Microcin_M, Colicin E1	Bottromycin	ComX4, Carocin_D	Thiopeptide	NRPS (6), NRP-metallophore (2)	Enterobactin, arylpolyene, T1PKS (2)

ESCHERICHIA COLI STR. UMB0900	Colicin_ E9	Carocin_D	Bottromycin	-	-	Thiopeptide	NRP-metallophore (2), NRPS (2)	T1PKS, NI-siderophore
ESCHERICHIA COLI STR. UMB0901	ComX4	Microcin	Bottromycin	Microcin_M	Colicin	Thiopeptide	NRP-metallophore (2), NRPS (3)	T3PKS
ESCHERICHIA COLI STR. UMB0727	Colicin (2)	Microcin	Bottromycin	Microcin_M	Colicin-10, Colicin E7	Thiopeptide	RiPP-like, NRPS (6), NRP-metallophore (2)	Enterobactin, arylpolyene, T1PKS (2)
FACKLAMIA HOMINIS STR. UMB0111	Lanthipeptide-Class-IV	-	-	-	-	-	Lasso -peptide	T3PKS
FACKLAMIA SP. STR. UMB0898	-	-	-	-	-	-	-	-
FINEGOLDIA MAGNA STR. UMB0115	Putative bacteriocin	Pneumolancidin	-	-	-	-	Ranthipeptide, Lanthipeptide-Class-II, NRPS, RiPP-like	RRE-containing
FUSOBACTERIUM NUCLEATUM STR. UMB0249	Sactipeptide	-	-	-	-	-	NRPS-like	-
GARDNERELLA VAGINALIS STR. UMB0061	Propioncin_ SM1	-	-	-	-	-	-	T3PKS
GARDNERELLA	Variacin	-	-	-	-	-	Lanthipeptide-	-

VAGINALIS STR. UMB0170							Class-II, RiPP-like	
GARDNERELLA VAGINALIS STR. UMB0912	-	-	-	-	-	-	-	-
GARDNERELLA VAGINALIS STR. UMB0913	-	-	-	-	-	-	-	-
GARDNERELLA VAGINALIS STR. UMB0682	-	-	-	-	-	-	-	-
GARDNERELLA VAGINALIS STR. UMB0264	-	-	-	-	-	-	-	-
GARDNERELLA VAGINALIS STR. UMB0233	-	-	-	-	-	-	-	-
GARDNERELLA VAGINALIS STR. UMB0032A	Propioncin_ SM1	-	-	-	-	-	-	T3PKS
GARDNERELLA VAGINALIS STR. UMB0386	Propioncin_ SM1	-	-	-	-	-	-	T3PKS
GARDNERELLA VAGINALIS STR. UMB0032B	Propioncin_ SM1	-	-	-	-	-	-	T3PKS
GARDNERELLA	Propioncin_	-	-	-	-	-	-	T3PKS

VAGINALIS STR. UMB0298	SM1							
GARDNERELLA VAGINALIS STR. UMB0768	Propioncin_ SM2	-	-	-	-	-	-	T3PKS
GARDNERELLA VAGINALIS STR. UMB0411	-	-	-	-	-	-	RiPP-like	-
GARDNERELLA VAGINALIS STR. UMB0663	Propioncin_ SM3	-	-	-	-	-	-	-
GARDNERELLA VAGINALIS STR. UMB1642	Variacin	-	-	-	-	-	Lanthipeptide-Class-II, RiPP-like	-
GARDNERELLA VAGINALIS STR. UMB0770	-	-	-	-	-	-	-	T3PKS
GARDNERELLA VAGINALIS STR. UMB1686	-	-	-	-	-	-	-	-
GARDNERELLA VAGINALIS STR. UMB0775	-	-	-	-	-	-	-	T3PKS
GARDNERELLA VAGINALIS STR. UMB0830	-	-	-	-	-	-	-	T3PKS
GARDNERELLA	-	-	-	-	-	-	-	T3PKS

VAGINALIS STR. UMB0833								
GARDNERELLA VAGINALIS STR. UMB1428	-	-	-	-	-	-	-	T3PKS
GEMELLA HAEMOLYSANS STR. UMB1385	-	-	-	-	-	-	-	T3PKS
GEMELLA SANGUINIS STR. UMB0186	Cytolysin_ClyL1	-	-	-	-	-	Lanthipeptide-Class-II, RiPP-like	-
GLOBICATELLA SANGUINIS STR. UMB0514	-	-	-	-	-	-	-	Betalactone
GORDONIA TERRAE STR. UMB0777	PoyA	Putative bacteriocin family protein	Sactipeptide	-	-	-	NRPS (7), Betalactone (2), RiPP-like	Terpene, NAPAA, NI-siderophore, Ectoine T1PKS, arylpolyene, redox- cofactor
HAEMOPHILUS PARAINFLUENZAE STR. UMB0748	-	-	-	-	-	-	RiPP-like	-
KLEBSIELLA PNEUMONIAE STR. UMB0411	-	-	-	-	-	-	-	-

KLEBSIELLA PNEUMONIAE STR. UMB0140	Bottromycin	-	-	-	-	Thiopeptide	NRP-metallophore, NRPS, RiPP-like	Redox-cofactor
KOCURIA RHIZOPHILA STR. UMB0131	-	-	-	-	-	-	-	Terpene, T3PKS, NI- siderophore, NAPAA, Betalactone
KYTOCOCCUS SCHROETERI STR. UMB1298	Propioncin_T1 (2)	-	-	-	-	-	RiPP-like	NAPAA, Terpene, NI-siderophore, Ectoine
LACTOBACILLUS CRISPATUS STR. UMB0803	Enterolysin_A	Bacteriocin_LS2chaina, ggmotif: ComC; bacteriocin_IIc	Bacteriocin helveticn_J	Helevticin_J	Penocin_A (2)	-	RiPP-like (4)	-
LACTOBACILLUS CRISPATUS STR. UMB0044	Enterolysin_A	Bacteriocin_LS2chaina, ggmotif: ComC; bacteriocin_IIc	Bacteriocin helveticn_J	Helevticin_J	Penocin_A (2)	-	RiPP-like (4)	-
LACTOBACILLUS CRISPATUS STR. UMB0085	ComC; L_biotic_typeA_bacteriocin IIc, ggmotif: ComC; bacteriocin	Bacteriocin_LS2chaina	Bovicin_225_peptide	Enterolysin_A	Bacteriocin helveticn_J	-	RiPP-like (3)	-
LACTOBACILLUS CRISPATUS STR.	Bacteriocin helveticn_J	Helevticin_ J	Helevticin_J	Bovicin_225_peptide	Enterolysin_A	-	LAP, RiPP-like	Furan

UMB0824								
LACTOBACILLUS CRISPATUS STR. UMB0040	Bacteriocin helveticn_J	Helevticin_ J	Bovicin_225_peptide	ComC; L_biotic_typeA_bacteriocin IIc, ggmotif: ComC; bacteriocin_IIc	Bacteriocin_LS2_chaina	-	RiPP-like (2)	-
LACTOBACILLUS CRISPATUS STR. UMB0040	Bacteriocin helveticn_J	Helevticin_ J	Bovicin_225_peptide	ComC; L_biotic_typeA_bacteriocin IIc, ggmotif: ComC; bacteriocin_IIc	Bacteriocin_LS2_chaina	-	RiPP-like (2)	-
LACTOBACILLUS CRISPATUS STR. UMB1398	Bacteriocin helveticn_J	Helevticin_ J	ComC; L_biotic_typeA_bacteriocin IIc, ggmotif: ComC; bacteriocin_IIc	Bacteriocin_LS2_chaina	Enterolysin_A	-	RiPP-like (2)	-
LACTOBACILLUS CRISPATUS STR.	Bacteriocin_LS2_chaina	Enterolysin_A	Enterolysin_A	Helevticin_J	-	-	RiPP-like (3)	-

UMB0054								
LACTOBACILLUS DELBRUECKII STR. UMB0003	Helevticin_ J	Helevticin_ J	Enterolysin_ A	-	-	-	-	-
LACTOBACILLUS FERMENTUM STR. UMB0187	Enterolysin_ A	-	-	-	-	-	-	Terpene
LACTOBACILLUS GASSERI STR. UMB0099	Acidiocin_LF221B (GassericinK7B), Gassericin_T	Bacteriocin_LS2chainb, Unidentified Bacteriocin	L_biotic_typeA: bacteriocin_IIc, ggmotif; Bacteriocin_IIc	Bacteriocin helveticn_J	Microcin_M	-	Gassericin_T, Gassericin-S	-
LACTOBACILLUS GASSERI STR. UMB0045	AcdB_acidocin_B	Bacteriocin helveticn_J	-	-	-	-	-	-
LACTOBACILLUS GASSERI STR. UMB0670	AcdB_acidocin_B	L_biotic_typeA; bacteriocin_IIc	Pedicion	Bacteriocin helveticn_J	-	-	-	-
LACTOBACILLUS GASSERI STR. UMB0045	AcdB_acidocin_B	Bacteriocin helveticn_J	-	-	-	-	-	-
LACTOBACILLUS GASSERI STR. UMB0056	Bacteriocin_LS2_chainb, Bacteriocin	Bacteriocin helveticn_J	-	-	-	-	Gassericin T, Gassericin-S	-
LACTOBACILLUS INERS STR. UMB0030	Mutacin_11 (MutacinH-29B)	Lanthipeptide-Class-II	-	-	-	-	RiPP-like, Lanthipeptide_Class-II	-
LACTOBACILLUS INERS	Mutacin_11	Lanthipeptide- Class-II	-	-	-	-	Lanthipeptide	-

STR. UMB0033	(MutacinH-29B)						- Class-II	
LACTOBACILLUS INERS STR. UMB1051	Mutacin_11 (MutacinH-29B)	Lanthipeptide-Class-IV	-	-	-	-	Mutacin II, Lanthipeptide-Class_V, Lanthipeptide_Class_III	-
LACTOBACILLUS JENSENII STR. UMB0007	-	-	-	-	-	-	NRPS	-
LACTOBACILLUS JENSENII STR. UMB0077	Enterolysin_A	-	-	-	-	-	NRPS	-
LACTOBACILLUS PONTIS STR. UMB0683	-	-	-	-	-	-	-	T3PKS, Furan
LACTOBACILLUS RHAMNOSUS STR. UMB0004	Carnocin_CP52	Enterocin_X_chain_beta	ggmotif; Bacteriocin_IIc	-	-	-	RiPP-like	T3PKS
LACTOBACILLUS VAGINALIS STR. UMB0388	Enterolysin_A	-	-	-	-	-	-	T3PKS
LECLERCIA ADECARBOXYLATA STR. UMB0660	Bottromycin	-	-	-	-	Thiopeptide	NRP-metallophore, NRPS	NI- siderophore, Butyrolactone, arylpolyene
MICROBACTERIUM SP. STR. UMB0228	-	-	-	-	-	-	LAP	Terpene, Betalactone, TPKS
MICROCOCCUS LUTEUS STR. UMB0189	-	-	-	-	-	-	-	NI-siderophore, NAPAA,

								Betalactone, Terpene, Ectoine, RRE-containing
MICROCOCCUS SP. STR. UMB0867	-	-	-	-	-	-	-	NI- siderophore, NAPAA, Betalactone, Terpene, Ectoine, RRE- containing
MICROCOCCUS SP. STR. UMB0031	-	-	-	-	-	-	-	NI-siderophore, NAPAA, Betalactone, Terpene, Ectoine, RRE-containing
MICROCOCCUS LUTEUS STR. UMB0189	-	-	-	-	-	-	-	NI-siderophore, NAPAA, Betalactone, Terpene, Ectoine, RRE-containing
MICROCOCCUS SP. STR. UMB0038	-	-	-	-	-	-	-	NI- siderophore, NAPAA, Betalactone, Terpene, Ectoine, RRE-

								containing
MICROCOCCUS LYLAE STR. UMB0955	Lanthipeptid e-Class-IV	-	-	-	-	-	Lanthipeptide-Class-III	Ectoine, Betalactone, NAPAA
MORAXELLA OSLOENSIS STR. UMB0416	-	-	-	-	-	-	-	Betalactone, NI-siderophore
MORGANELLA MORGANII STR. UMB1297	Bottromycin	Colicin_E6	Microcin	-	-	Thiopepti de	RiPP-like, NRPS	Betalactone, T1PKS
NEISSERIA PERFLAVA STR. UMB0023	-	-	-	-	-	-	-	Terpene, Arylpolyene (2), hserlactone, Resorcinol
NEISSERIA PERFLAVA STR. UMB0210	-	-	-	-	-	-	-	Terpene, Arylpolyene (2), hserlactone, Resorcinol
NEISSERIA SICCA STR. UMB0321	-	-	-	-	-	-	-	Terpene, hserlactone
NOSOCOMIICOCCUS MASSILIENSIS STR. UMB0959	Lanthipeptid e-Class-I	-	-	-	-	-	Lanthipeptide-Class-I	-
NOSOCOMIICOCCUS MASSILIENSIS STR. UMB0959	Lanthipeptid e-Class-I	-	-	-	-	-	Lanthipeptide-Class-I	-

OLIGELLA URETHRALIS STR. UMB0345	-	-	-	-	-	-	-	Betalactone, Terpene, NI_siderophore
PREVOTELLA BUCCALIS STR. UMB0536	-	-	-	-	-	-	-	RRE-containing
PREVOTELLA TIMONENSIS STR. UMB0818	LAPs	Sactipeptide	-	-	-	-	LAP	RRE-containing
PROPIONIBACTERIUM ACNES STR. UMB0211	TP-1161	TP-1161	-	-	-	Thiopeptide, Cutimycin	RiPP-like, NRPS (2)	-
PROTEUS MIRABILIS STR. UMB0315	Bottromycin	Colicin	-	-	-	Thiopeptide	RiPP-like, NRPS	Betalactone, NI-siderophore, T1PKS, Ladderane
PSEUDOMONAS AERUGINOSA STR. UMB0740	Bottromycin	Colicin-10	Pyocin_AP41_subunit	Pyocin_S1	-	Thiopeptide	Pyocyanine (2), NRPS-like (2), NRPS (7)	Redox-cofactor, hserlactone, Betalactone, NAGGN, opine-like-metallophore, pyochelin
ROTHIA DENTOCARIOSA STR. UMB0083	LAPs	Lichenicidin VK21A1	ggmotif; LE-DUF; LE-LAC481;	MA-2PEPA	L_biotic_typeA	-	LAP, Lanthipeptide-Class_II	Enterobactin, RRE-containing, Butyrolactone

			LE-MER+2PEP; L_biotic_typeA;					
ROTHIA MUCILAGINOSA STR. UMB0024	Geobacillin	Sactipeptide	-	-	-	-	-	Enterobactin
STAPHYLOCOCCUS AUREUS STR. UMB0910	Putative bacteriocin	Sactipeptide	Auto-inducing-peptide- III	Delta-lysin	-	-	RiPP-like, NRPS	Staphyloferrin A, Staphyloferrin B, Staphylopine cyclic-lactone-autoinducer, Terpene, T3PKS
STAPHYLOCOCCUS HOMINIS STR. UMB0272	Auto_inducing_peptide_II	Sactipeptide	Amylocyclin	-	-	-	RiPP-like (2)	Cyclic-lactone-autoinducer, NI-siderophore, T3PKS
STAPHYLOCOCCUS SP. STR. UMB0328	Auto_inducing_peptide_II	Delta-lysinI (2)	Lanthipeptide- Class-II	Sactipeptide	Warnericin_RC	-	Lanthipeptide-Class-II	Staphylopine, Staphyloferrin A, T3PKs, Terpene, cyclic lactone autoinducer
STAPHYLOCOCCUS PETTENKOFERI STR. UMB0834	Auto_inducing_peptide_II	Sactipeptide	Zoocin_A	-	-	-	Epipeptide	Terpene, NI_siderophore (2), T3PKS,

								cyclic-lactone-autoinducer
STREPTOCOCCUS SP. STR. UMB0039	-	-	-	-	-	-	-	RRE-containing
STREPTOCOCCUS SP. STR. UMB0767	Zoocin_A	-	-	-	-	-	-	Arylpolyene, T3PKS
STREPTOCOCCUS AGALACTIAE STR. UMB0776	Zoocin_A	-	-	-	-	-	-	Arylpolyene, T3PKS
STREPTOCOCCUS AGALACTIAE STR. UMB0049	Zoocin_A	-	-	-	-	-	-	Arylpolyene, T3PKS
STREPTOCOCCUS ANGINOSUS STR. UMB0252	-	-	-	-	-	-	-	-
STREPTOCOCCUS ANGINOSUS STR. UMB0839	Zoocin_A	-	-	-	-	-	RiPP-like	T3PKS
STREPTOCOCCUS ANGINOSUS STR. UMB0820								T3PKS
STREPTOCOCCUS ANGINOSUS STR. UMB0329	-	-	-	-	-	-	RiPP-like	T3PKS
STREPTOCOCCUS ANGINOSUS STR. UMB0220	-	-	-	-	-	-	RiPP-like	T3PKS

STREPTOCOCCUS ANGINOSUS STR. UMB0434	-	-	-	-	-	-	RiPP-like	T3PKS
STREPTOCOCCUS ANGINOSUS STR. UMB0050	-	-	-	-	-	-	RiPP-like	T3PKS
STREPTOCOCCUS ANGINOSUS STR. UMB0142	-	-	-	-	-	-	RiPP-like	T3PKS
STREPTOCOCCUS DENTISANI STR. UMB0832	Sactipeptide	-	-	-	-	-	RiPP-like, NRPS	T3PKS
STREPTOCOCCUS DENTISANI STR. UMB0008	Acidiocin_LF221B (GassericinK7B), Gassericin_T	-	-	-	-	-	RiPP-like (2)	T3PKS
STREPTOCOCCUS DENTISANI STR. UMB0029	LAPs	Lichenicidin VK21A2_ (Lichenicidin_A2)	Pneumolancidin (2)	Sactipeptide (2)	Thusin_alpha	Thiopeptide	Lanthipeptide-Class-II, RiPP-like (2), RaS-RiPP	Furan
STREPTOCOCCUS DENTISANI STR. UMB0079	Pneumolancidin (3)	-	-	-	-	-	RiPP-like (2)	T3PKS
STREPTOCOCCUS MITIS STR. UMB1341	-	-	-	-	-	-	RiPP-like	T3PKS
STREPTOCOCCUS	Pneumolanci	-	-	-	-	-	RiPP-like (2)	T3PKS

DENTISANI STR. UMB0079	din (3)							
STREPTOCOCCUS PARASANGUINI STR. UMB0216	Sactipeptide	-	-	-	-	-	-	-
STREPTOCOCCUS MACEDONICUS STR. UMB0733	Ubericin_A	Bovicin_225_peptide (4)	Nisin_U	-	-	-	Lanthipeptide-Class-I, RiPP-like	T3PKS
STREPTOCOCCUS SALIVARIUS STR. UMB0051	Salvaricin_A5	Thermophillin_A	Bacteriocin_J46 (2), McdA1	BIpI	-	-	Salvaricin A, Suicin 65, RiPP-like (2)	T3PKS
STREPTOCOCCUS SALIVARIUS STR. UMB0028	Streptin	-	-	-	-	-	Streptin	T3PkS
TRUEPERELLA BERNARDIAE STR. UMB0116	Sactipeptide	-	-	-	-	-	-	-
VARIBACULUM CAMBRIENSE STR. UMB0744	Sactipeptide	-	-	-	-	-	RiPP-like	-
VARIBACULUM CAMBRIENSE STR. UMB0796	Sactipeptide	-	-	-	-	-	RiPP-like	-
VEILLONELLA PARVULA STR. UMB0371	-	-	-	-	-	-	Ranthipeptide	-
YOKENELLA REGENSBURGEI STR.	Bottromycin	-	-	-	-	Thiopeptide	Enterobactin, NRP-	Butyrolactone, Arylpolyene, NI-

UMB0819	metallophore	siderophore

Supplementary Table 2. Urobiome strains which encode putative bacteriocin gene cluster(s) with surrounding accessory genes present

BAGEL4 AOIs	Genome name	BLASTP result	Query cover	% identity	Alignment EMBOSS	Accession number
ACIDIOCIN_LF221B(GASSERICINK7B)	*Streptococcus dentisani* str. UMB0008	Blp family class II bacteriocin [*Streptococcus oralis*]	100%	100%	100%	WP_049537864.1
ACIDOCIN_LF22IB(GASSERICINK7B)	*Lactobacillus gasseri* str. UMB0099	Blp family class II bacteriocin [*Lactobacillus*]	100%	100%	100%	WP_003649213.1
AMYLOCYCLIN*	*Staphylococcus hominis* str. UMB0272	Uberolysin/carnocyclin family circular bacteriocin [*Staphylococcus hominis*]	100%	100%	89%	WP_101804450.1
BACTERIOCIN*	*Lactobacillus gasseri* str. UMB0056	Bacteriocin [*Lactobacillus*]	100%	100%	100%	WP_049160225.1
BACTERIOCIN_LS2CHAINA*	*Lactobacillus crispatus* str. UMB0040	Blp family class II bacteriocin [*Lactobacillus amylovorus*]	100%	99%	99%	WP_013642488.1
BACTERIOCIN_LS2CHAINA*	*Lactobacillus crispatus* str. UMB0803	Blp family class II bacteriocin [*Lactobacillus amylovorus*]	100%	99%	99%	WP_013642488.1
BACTERIOCIN_LS2CHAINA*	*Lactobacillus crispatus* str. UMB0085	Blp family class II bacteriocin [*Lactobacillus crispatus*]	100%	99%	99%	WP_013642488.1
BACTERIOCIN_LS2CHAINA*	*Lactobacillus crispatus* str.	Blp family class II bacteriocin [*Lactobacillus crispatus*]	100%	99%	99%	WP_013642488.1

	UMB0044					
BACTERIOCIN_LS2CHAINA*	*Lactobacillus crispatus* str. UMB1398	Blp family class II bacteriocin [*Lactobacillus crispatus*]	100%	99%	99%	WP_013642488.1
BACTERIOCIN_LS2CHAINA	*Lactobacillus crispatus* str. UMB0054	Blp family class II bacteriocin [*Lactobacillus crispatus*]	100%	100%	100%	WP_005726423.1
BACTERIOCIN_LS2CHAINB*	*Lactobacillus gasseri* str. UMB0056	Blp family class II bacteriocin [*Ligilactobacillus salivarius*]	97%	47.76%	43.8%	WP_032495430.1
BACTERIOCIN_LS2CHAINB*	*Lactobacillus gasseri* str. UMB0099	Blp family class II bacteriocin [*Ligilactobacillus salivarius*]	97%	47.76%	43.8%	WP_032495430.1
BIPI*	*Streptococcus salivarius* str. UMB0051	Class IIb bacteriocin, lactobin A/cerein 7B family [*Streptococcus*]	87%	100%	87.1%	WP_225791565.1
BIPM	*Streptococcus dentisani* str. UMB0008	Two-peptide bacteriocin subunit BlpM [*Streptococcus*]	100%	100%	100%	WP_000379951.1
BIPM	*Streptococcus dentisani* str. UMB0008	Blp family class II bacteriocin [*Streptococcus oralis*]	100%	100%	100%	WP_101800376.1
BIPN	*Streptococcus dentisani* str. UMB0008	Blp family class II bacteriocin [Bacteria]	100%	100%	100%	WP_001099737.1
BIPU*	*Streptococcus dentisani* str.	MULTISPECIES: bacteriocin class II family protein [Bacteria]	100%	100%	100%	WP_070800393.1

	UMB0008					
BLPC*	*Streptococcus dentisani* str. UMB0008	Quorum-sensing system pheromone BlpC [*Streptococcus oralis*]	100%	100%	100%	WP_101800373.1
BOVICIN_225_PEPTIDE*	*Streptococcus macedonicus* str. UMB0733	Bacteriocin [*Streptococcus*]	74%	66.27%	49.1%	WP_039694458.1
BOVICIN_225_PEPTIDE*	*Streptococcus macedonicus* str. UMB0733	Garvicin Q family class II bacteriocin [*Streptococcus gallolyticus*]	100%	80%	43.9%	WP_039694458.1
BOVICIN_225_PEPTIDE*	*Streptococcus macedonicus* str. UMB0733	Garvicin Q family class II bacteriocin [*Streptococcus gallolyticus*]	60%	80%	32.4%	WP_039694458.1
BOVICIN_225_PEPTIDE*	*Streptococcus macedonicus* str. UMB0733	Bacteriocin class II with double glycine leader peptide [*Streptococcus infantarius subsp. Infantarius*]	100%	95.9%	95.9%	MCO4620482.1
CARNOCIN_CP52*	*Lactobacillus rhamnosus* str. UMB0004	Bacteriocin immunity protein [*Lacticaseibacillus rhamnosus*]	100%	100%	100%	WP_047678371.1
COLICIN	*Citrobacter murliniae* str. UMB1094	Type VI secretion system tube protein TssD [*Citrobacter freundii*]	100%	100%	100%	WP_102190731.1
COLICIN*	*Escherichia coli str.* UMB0901	Pyocin-S2 [*Escherichia coli*]	100%	100%	100%	CAF4718426.1
COLICIN*	*Escherichia coli* str. UMB0727	Pyocin-S2 [*Escherichia coli*]	100%	100%	100%	CAF4718426.1

COLICIN*	*Proteus mirabilis* str. UMB0315	Colicin-E2 [*Proteus mirabilis*]	99%	99%	99.1%	AWF41001.1
COLICIN-10	*Escherichia coli* str. UMB0727	Colicin-like pore-forming protein [*Escherichia coli*]	100%	100%	100%	WP_016232721.1
COLICIN_E7	*Escherichia coli* str. UMB0727	Colicin-like bacteriocin tRNase domain-containing protein [*Escherichia coli*]	100%	100%	100%	WP_042021075.1
COLICIN_E9*	*Escherichia coli* str. UMB0789	Type VI secretion system tube protein TssD [*Escherichia coli*]	100%	100%	99.8%	WP_000502506.1
COLICIN_E9*	*Escherichia coli* str. UMB0900	Colicin [*Escherichia coli*]	100%	100%	99.7%	STP22082.1
COLICIN_E6	*Morganella morganii* str. UMB1297	Colicin E3/pyocin S6 family cytotoxin [*Morganella morganii*]	100%	100%	100%	WP_180801870.1
COMC; L_BIOTIC_TYPEA; BACTERIOCIN_IIC*	*Lactobacillus crispatus* str. UMB0040	Hypothetical protein [*Lactobacillus*]	100%	100%	100%	WP_005720990.1
COMC; L_BIOTIC_TYPEA; BACTERIOCIN_IIC*	*Lactobacillus crispatus* str. UMB0085	Bacteriocin [*Lactobacillus crispatus*]	100%	100%	100%	WP_005720990.1
COMC; L_BIOTIC_TYPEA; BACTERIOCIN_IIC*	*Lactobacillus crispatus* str. UMB1398	Hypothetical protein [*Lactobacillus*]	100%	100%	100%	WP_005720990.1
COMC; L_BIOTIC_TYPEA; BACTERIOCIN_IIC*	*Streptococcus macedonicus* str. UMB0733	Bacteriocin [*Streptococcus gallolyticus*]	100%	78%	78%	WP_114317773.1
ENTEROCIN_X_CHAIN_	*Lactobacillus*	Class IIb bacteriocin, lactobin A/cerein 7B family	100%	100%	100%	WP_0476

BETA*	*rhamnosus* str. UMB0004	[*Lacticaseibacillus rhamnosus*]				78355.1
GASSERICIN_T	*Lactobacillus gasseri* str. UMB0099	Blp family class II bacteriocin [*Lactobacillus paragasseri*]	100%	100%	100%	WP_0491 59833.1
GGMOTIF; BACTERIOCIN_IIC;	*Lactobacillus gasseri* str. UMB0099	Blp family class II bacteriocin [*Lactobacillus*]	100%	100%	100%	WP_1018 90487.1
GGMOTIF; BACTERIOCIN_IIC*	*Lactobacillus rhamnosus* str. UMB0004	Bacteriocin [*Lacticaseibacillus rhamnosus*]	100%	100%	100%	WP_0491 71132.1
GGMOTIF; COMC; BACTERIOCIN_IIC*	*Lactobacillus crispatus* str. UMB0803	Bacteriocin [*Lactobacillus crispatus*]	100%	98%	98%	MCT788 7040.1
GGMOTIF; COMC; BACTERIOCIN_IIC*	*Lactobacillus crispatus* str. UMB0040	Bacteriocin [*Lactobacillus crispatus*]	100%	98%	98%	MCT788 7040.1
GGMOTIF; COMC; BACTERIOCIN_IIC*	*Lactobacillus crispatus* str. UMB0085	Bacteriocin [*Lactobacillus crispatus*]	100%	98%	98%	MCT788 7040.1
GGMOTIF; COMC; BACTERIOCIN_IIC*	*Lactobacillus crispatus* str. UMB0044	Bacteriocin [*Lactobacillus crispatus]*	100%	98%	98%	MCT788 7040.1
GGMOTIF; COMC; BACTERIOCIN_IIC*	*Lactobacillus crispatus* str. UMB1398	Bacteriocin [*Lactobacillus crispatus*]	100%	98%	98%	MCT788 7040.1
L_BIOTIC_TYPEA:	*Lactobacillus gasseri*	Lactacin F inducer peptide precursor [*Lactobacillus*	100%	84%	92%	WP_2603

BACTERIOCIN_IIC*	str. UMB0099	*johnsonii*]				07981.1
L_BIOTIC_TYPEA; BACTERIOCIN_IIC*	*Lactobacillus gasseri* str. UMB0670	Lactacin F inducer peptide precursor *[Lactobacillus gasseri*]	98%	100%	98%	WP_225792992.1
LAPS (SONORENSIN)*	*Bacillus cibi str.* UMB0893	Heterocycloanthracin/sonorensin family bacteriocin [*Bacillus sp*. UMB0893]	73%	100%	73.3%	PLR66119.1
LICHENICIDINVK21A1*	*Aerococcus sanguinicola* str. UMB0139	Plantaricin C family lantibiotic [*Aerococcus sanguinicola*]	100%	100%	100%	WP_101603837.1
LICHENICIDINVK21A2_(LICHENICIDIN_A2)*	*Streptococcus dentisani* str. UMB0029	Class II lanthipeptide, LchA2/BrtA2 family [*Streptococcus sp*. UMB0029]	100%	100%	87.5%	WP_102210124.1
MCDA1	*Streptococcus salivarius* str. UMB0051	Lacticin 481 family lantibiotic [*Streptococcus*]	100%	100%	100%	WP_080702563.1
MICROCIN_H47_(MCCH47)	*Escherichia coli* str. UMB0789	MchB protein [*Escherichia coli* CFT073]	100%	100%	100%	WP_080702563.1
MICROCIN_M	*Escherichia coli* str. UMB0789	Microcin McmA [*Escherichia*]	100%	100%	100%	WP_001318125.1
MICROCIN_M*	*Lactobacillus gasseri* str. UMB0099	Bacteriocin [*Lactobacillus*]	100%	100%	100%	WP_101890486.1
MUTACIN_11(MUTACINH-29B)	*Lactobacillus gasseri* str. UMB1051	Lacticin 481 family lantibiotic [*Lactobacillus iners*]	100%	100%	100%	WP_276508305.1
MUTACIN_11(MUTACINH-29B)	*Gardnerella vaginalis* str. UMB0033	Type A2 lanthipeptide [*Lactobacillus iners*]	100%	100%	100%	WP_240398376.1
MUTACIN_11(MUTACI	*Lactobacillus iners*	Type A2 lanthipeptide [*Lactobacillus iners*]	100%	100%	63.1%	WP_2403

NH-29B)*	str. UMB0030					98376.1
NISIN_U*	*Streptococcus macedonicus* str. UMB0733	Gallidermin/nisin family lantibiotic [*Streptococcus suis*]	100%	100%	75%	WP_228478826.1
PEDIOCIN*	*Lactobacillus gasseri* str. UMB0099	Bacteriocin immunity protein [*Lactobacillus*]	100%	100%	100%	WP_101890489.1
PEDIOCIN*	*Lactobacillus gasseri* str. UMB0670	Pediocin immunity protein PedB *[Lactobacillus gasseri* ATCC 33323 = JCM 1131]	100%	100%	89%	ABJ59938.1
PENOCIN_A*	*Lactobacillus crispatus* str. UMB0044	TIGR04139 family peptide modification target [*Lactobacillus crispatus*]	100%	100%	96%	AZR16416.1
PENOCIN_A*	*Lactobacillus crispatus* str. UMB0803	Hypothetical protein [*Lactobacillus crispatus*]	98%	40.62 %	34.7%	WP_154626382.1
PLANTARICIN C FAMILY LANTIBIOTIC*	*Staphylococcus sp.* str. UMB0328	Plantaricin C family lantibiotic [*Staphylococcus*]	100%	100%	100%	WP_104681709.1
PNEUMOLANCIDIN	*Finegoldia magna* str. UMB0115	Salivaricin M family lantibiotic [*Finegoldia magna*]	100%	100%	100%	WP_094206507.1
PUTATIVE BACTERIOCIN*	*Streptococcus dentisani* str. UMB0008	ComC/BlpC family leader-containing pheromone/bacteriocin [*Streptococcus*]	100%	100%	100%	WP_070800397.1
PUTATIVE BACTERIOCIN*	*Lactobacillus crispatus* str. UMB0085	Lactococcin 972 family bacteriocin [*Lactobacillus*]	100%	100%	100%	WP_061205069.1
PUTATIVE	*Finegoldia magna* str.	Lactococcin 972 family bacteriocin [*Finegoldia*]	100%	100%	95.5%	WP_0028

BACTERIOCIN*	UMB0115					40185.1
PYOCIN_AP41_SUBUNIT	*Pseudomonas aeruginosa* str. UMB0740	Pyocin_S1 [*Pseudomonas*]	100%	100%	100%	WP_003145451.1
PYOCIN_S1	*Pseudomonas aeruginosa* str. UMB0740	S-type pyocin domain-containing protein [*Pseudomonas aeruginosa*]	100%	100%	100%	WP_023088120.1
SALVARICIN_A5	*Streptococcus salivarius* str. UMB0051	Type A2 lanthipeptide [*Streptococcus salivarius*]	100%	100%	100%	WP_101799750.1
STREPTIN	*Streptococcus salivarius* str. UMB0028	Lantibiotic streptin [*Streptococcus sanguinis*]	100%	100%	100%	WP_002917611.1
SUBTILIN*	*Rothia mucilaginosa* str. UMB0024	Gallidermin/nisin family lantibiotic [*Actinomyces sp*. ICM58]	100%	83.54%	83.5%	WP_081495980.1
THERMOPHILLIN_A	*Streptococcus salivarius* str. UMB0051	Blp family class II bacteriocin [*Streptococcus*]	100%	100%	100%	WP_048674859.1
THUSIN_ALPHA	*Streptococcus dentisani* str. UMB0029	Lichenicidin alpha family lanthipeptide [*Streptococcus sp*. UMB0029]	100%	100%	100%	WP_102210125.1
UBERICIN_A*	*Streptococcus macedonicus* str. UMB0733	Blp family class II bacteriocin [*Streptococcus*]	93%	100%	93.5%	WP_003066580.1
UNIDENTIFIED	*Lactobacillus gasseri*	Bacteriocin [*Lactobacillus*]	100%	100%	100%	WP_0491

BACTERIOCIN*	str. UMB0099					60225.1
VARIACIN (17.2 BACTERIOCIN_J46)	*Gardnerella vaginalis* str. UMB1642	Lacticin 481 family lantibiotic [*Gardnerella*]	100%	100%	100%	WP_0041 11352.1
VARIACIN (17.2 BACTERIOCIN_J46)	*Gardnerella vaginalis* str. UMB0170	Lacticin 481 family lantibiotic [*Gardnerella*]	100%	100%	100%	WP_0041 11352.2
VARIACIN (17.2 BACTERIOCIN_J46)	*Streptococcus salivarius* str. UMB0051	Lacticin 481 family lantibiotic [*Streptococcus*]	100%	100%	100%	WP_0376 01371.1
VARIACIN (17.2 BACTERIOCIN_J46)*	*Streptococcus salivarius* str. UMB0051	Lacticin 481 family lantibiotic [*Streptococcus salivarius*]	100%	100%	98%	WP_0376 01366.1

Putative bacteriocin hits are presented with their closest homologues as identified through BLASTP analysis and alignment using EBI EMBOSS needle, with an * representing the bacteriocins that are potentially novel by either differing by two or more amino acids or matching to a reported but previously uncharacterised bacteriocin.
